# Epithelial-myoepithelial carcinoma originating from a minor salivary gland in the nasal septum: A case report and literature review

**DOI:** 10.1097/MD.0000000000019072

**Published:** 2020-01-31

**Authors:** Young Sub Lee, Sun Mok Ha, Seung Won Paik, Hui Joon Yang, Hyun Jong Jeon, Dong-Joon Park, Chi Sang Hwang

**Affiliations:** a Department of Otorhinolaryngology, Yonsei University Wonju College of Medicine; b Department of Biomedical Laboratory Science, Yonsei University Wonju College of Health Science, Wonju, South Korea.

**Keywords:** epithelial-myoepithelial carcinoma, minor salivary gland, nasal septum, sinonasal region, surgical treatment

## Abstract

**Rationale:**

Epithelial-myoepithelial carcinoma is an extremely rare, malignant neoplasm that occurs most frequently in the major salivary glands and accounts for approximately 1% of all salivary gland neoplasms. Few reports have described the presence of epithelial-myoepithelial carcinoma in the sinonasal region; hence, the treatment guideline and prognosis remain unclear.

**Patient concerns:**

We reported a case of a 75-year-old woman with complaint of nasal obstruction and frequent epistaxis for 3 years. During the nasal endoscopic examination, a mass in the left nasal cavity originating from the left nasal septum that caused bleeding on touch was observed.

**Diagnoses:**

A diagnosis of epithelial-myoepithelial carcinoma was made based on the features of histopathology and immunohistochemistry of the surgical specimens. The patient was treated by surgical removal of the septal mass using the endonasal endoscopic approach.

**Outcomes:**

In the **s**erial follow-up paranasal sinus imaging and endoscopic inspection, evidence of recurrence was absent for 18 months after surgery.

**Lessons:**

This report highlights a case of epithelial-myoepithelial carcinoma originating from a minor salivary gland in the nasal septum, one of the most unusual locations. Diagnosis of epithelial-myoepithelial carcinoma should be made based on the findings of immunohistochemistry of the operative specimen. Clinicians should consider complete surgical resection as the effective treatment of choice.

## 1 Introduction

Epithelial-myoepithelial carcinoma is a histopathological term used to describe the biphasic pattern of clear staining of the myoepithelial cells surrounding variable proportions of the ducts with an epithelial lining.^[1]^ It is commonly found in the major salivary glands, including the parotid and submandibular glands.^[2,3]^ According to the literature, the sinonasal region is a rare location for the development of epithelial-myoepithelial carcinoma; however, the presence of minor salivary glands in the sinonasal mucosa may allow the occurrence of epithelial-myoepithelial carcinoma. The largest cohort study to date, which included 468 patients with epithelial-myoepithelial carcinoma, identified only 18 cases (3.8%) of the tumor in the sinonasal region.^[2]^ Moreover, epithelial-myoepithelial carcinoma in the nasal septum is extremely rare, and to the best of our knowledge, only one case has been reported in the English literature.^[4]^

Due to its rare occurrence, the pathophysiology, clinical features, and optimal management strategies of epithelial-myoepithelial carcinoma have not been fully described, and the relevant literature mostly comprises case reports. We recently encountered the case of a patient with epithelial-myoepithelial carcinoma originating from a minor salivary gland in the nasal septum, which was successfully treated by an endonasal endoscopic approach. In this case report, we described the clinical, histopathological, and immunohistochemical characteristics of this rare entity and performed a literature review of relevant cases.

## 2 Case report

**Figure 1:**
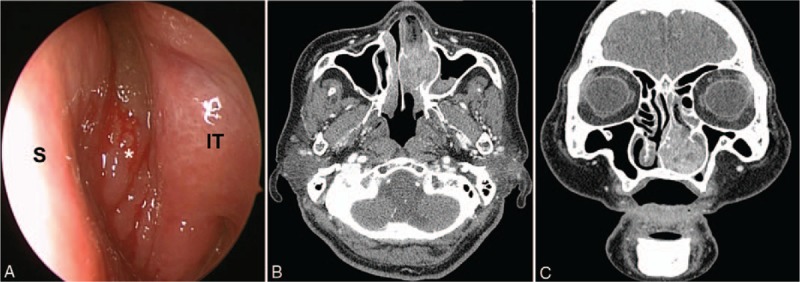
Preoperative imaging. (A) Nasoendoscopy reveals a polypoid mass (asterisk) causing bleeding on touch in the left nasal cavity. (B) Axial and (C) coronal contrast-enhanced computed tomography of the paranasal sinuses shows a 3.7-cm enhancing and expanding lesion in the left nasal cavity with accompanied remodeling of the nasal septum and inferior turbinate. IT = inferior turbinate, S = septum.

A 75-year-old woman presented at our emergency department with a complaint of progressive epistaxis. The patient had a 3-year history of nasal obstruction and intermittent epistaxis and absence of specific underlying systemic disease or trauma in her medical history. In the nasal endoscopic examination at the time of visit to our hospital, a hemorrhagic mass filling up the left nasal cavity without any ulceration was observed (Fig. 1A). In the laboratory tests, unremarkable findings were noted, which did not suggest a diagnosis of the nasal mass. A 3.7 × 2.5-cm bulging mass with heterogeneous enhancement in the left nasal cavity, and blurred boundary of the paranasal sinus was observed in the computed tomography (CT) scans (Fig. 1B and C). Destruction of the bone of the nasal floor or medial wall of the maxillary sinus was not evident.

**Figure 2:**
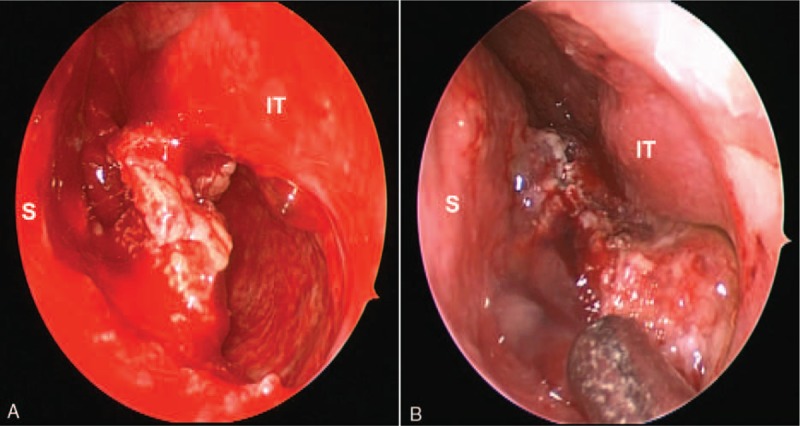
Intraoperative view. (A) Intraoperatively, the floor and lateral wall of the nasal cavity are intact. Remodeling of the inferior turbinate by the tumor is observed throughout the inferior meatus, but tumor invasion is not evident. (B) The tumor originated from the nasal septum is observed, and removal of the affected septal mucosa along with the tumor is performed. IT = inferior turbinate, S = septum.

A presumptive diagnosis of malignancy was made based on the aggressive appearance, despite the lack of significant cervical adenopathy. Due to active bleeding caused by the mass in the nasal cavity, diagnosis through biopsy of the mass preoperatively was not possible. Therefore, excisional biopsy and surgical resection via endonasal endoscopic approach were performed in the patient under general anesthesia. Intraoperatively, the nasal floor and lateral nasal wall were free from attachment to the tumor, but the inferior turbinate was remodeled by the tumor (Fig. 2A); possible origin of the tumor at the nasal septum without infiltration of the septal cartilage was observed (Fig. 2B). Mitotic figures in the tumor cells were observed in the intraoperative frozen section biopsy; however, differentiating between the presence of benign or malignant tumor was difficult. Subsequently, continuous peeling of the affected septal mucosa and finally, complete extirpation of the lesion was performed by the surgeon.

**Figure 3:**
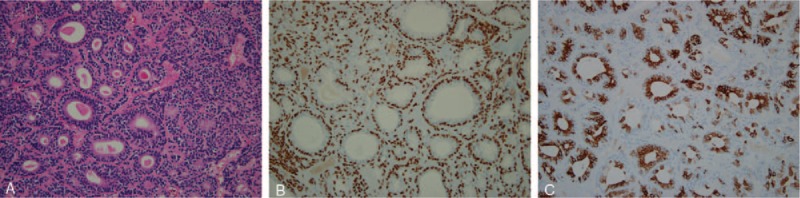
Histopathologic features of the tumor. (A) The tumor cells are composed of variable sized ductal structures lined with inner polymorphic epithelial cells surrounded by outer myoepithelial cells (hematoxylin and eosin staining, × 200). In immunohistochemical analysis, (B) the neoplastic myoepithelial cells (ductal cells of the outer layer) show reactivity to p63 (× 200), and (C) the luminal epithelial cells (inner layer) show strong positive immunoreactivity to cytokeratin-7 (× 200); hence, epithelial-myoepithelial carcinoma is diagnosed.

**Figure 4:**
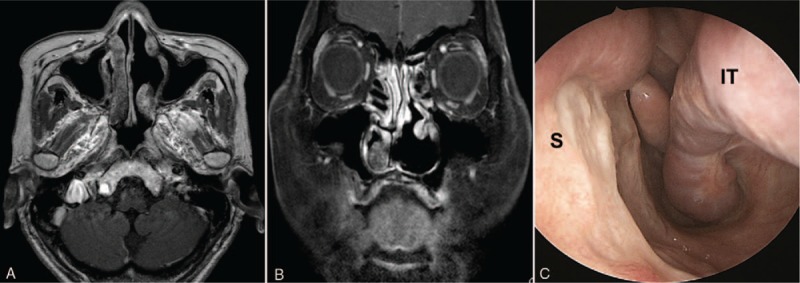
Follow-up paranasal sinus imaging and nasoendoscopic view at 18 months after surgery. (A) Axial and (B) coronal magnetic resonance imaging of the paranasal sinuses show a clear nasal cavity without recurrence. (C) Nasoendoscopy reveals complete resolution of the tumor and recovery of the septal mucosa. IT = inferior turbinate, S = septum.

In the final pathology report, two components of the tumor cells including the luminal ductal inner epithelial cells with nuclear polymorphism and peripheral outer myoepithelial cells were revealed (Fig. 3A). The results of biphasic differentiation were confirmed by immunohistochemical staining: positive for p63 and S-100 in the surrounding myoepithelial cells, and intense positive for epithelial markers, such as cytokeratin-7 in the luminal tumor cells (Fig. 3B and C, respectively). Based on these results, the mass was diagnosed as an epithelial-myoepithelial carcinoma of the nasal septum. The Ki-67 labeling index (proliferation index) of 3.5% was obtained. No evidence of regional or distant metastases through 2-Deoxy-2-(^18^F)-fluoro-D-glucose (FDG) positron emission tomography (PET)/CT was found postoperatively. The surgical specimens were fragmented such that the margins could not be properly evaluated; thus, radiotherapy for the locoregional area was recommended as adjuvant therapy. However, additional therapy could not be performed due to patient refusal. In the follow-up of the patient for 18 months after surgery, no recurrence was observed, and ongoing follow-up is being conducted (Fig. 4). This study was approved by the institutional review board of the Yonsei University Wonju College of Medicine. Informed consent was given by the patient.

## 3 Discussion

Epithelial-myoepithelial carcinoma represents approximately 1% of all salivary gland neoplasms.^[5,6]^ It is typically regarded as low-to-intermediate grade malignancy and is associated with favorable prognosis. Regional lymph node metastasis and distant metastasis are uncommon and occur at less than 5% each, in the recent population-based analysis^[2]^; moreover, the 5- and 10-year overall survival rates are 72.7 and 59.5%, respectively.^[2]^ In the previous studies, the majority (> 80%) of patients were > 50 years-old and there was a female predominance (1.5∼6:1),^[2,5]^ which is in agreement with characteristics of our patient.

Clinical manifestations of epithelial-myoepithelial carcinoma are usually nonspecific and variable depending on the site of origin and extent of each case. Tumors involving the nasal cavity may cause various symptoms such as facial pain, swelling, headache, epiphora, epistaxis, nasal obstruction, or rhinorrhea.^[1,5,7,8]^ In our case, the lesion occupied the nasal cavity and led to bleeding on touch, which caused the patient to complain of nasal obstruction and severe progressive epistaxis.

The diagnosis of sinonasal epithelial-myoepithelial carcinoma is rarely suspected before results of immunohistochemical staining of the surgical specimen are obtained. CT examination may reveal a heterogeneously enhancing soft tissue mass of the involved nasal or paranasal sinus with destruction of the adjacent structures, however, such finding of CT is not specific to epithelial-myoepithelial carcinoma. In addition, epithelial-myoepithelial carcinoma may be without accompanied tissue destruction as in our case. Recent reports have indicated that epithelial-myoepithelial carcinoma showed insignificant glucose uptake in PET/CT, which may be associated with the low-grade malignant potential of the epithelial-myoepithelial carcinoma.^[9,10]^ Therefore, preoperative diagnosis based on imaging alone is a challenge. In the radiological differential diagnosis, investigators should consider mucocele, invasive fungal sinusitis, or other neoplasms.

The final diagnosis of epithelial-myoepithelial carcinoma should be made based on conventional optical microscopy and immunohistochemistry. The histological differential diagnosis of epithelial-myoepithelial carcinoma includes all other salivary gland neoplasms such as pleomorphic adenoma, myoepithelial carcinoma, adenoid cystic carcinoma, acinic cell carcinoma, mucoepidermoid carcinoma, and metastatic renal cell carcinoma.^[11]^ The main histological features are biphasic tubular neoplasm composed of variable sized duct-like structures lined with epithelial cells in the inner layer of the lumen and myoepithelial cells in the surrounding outer layer.^[1]^ Compared with the low specificity of radiologic imaging, immunohistochemistry was useful to distinguish epithelial-myoepithelial carcinoma by discriminating the unique characteristics of the epithelial and myoepithelial phenotypes: positive for cytokeratin-7 and epithelial membrane antigen in the luminal epithelial cells, and positive for calponin, smooth muscle actin, S-100 protein, and p63 protein in the abluminal myoepithelial cells.^[12]^ In our case, we observed positive staining for p63 and S-100 in the outer myoepithelial cells and with cytokeratin-7 in the inner epithelial cells.

Currently, there is no consensus regarding optimal treatment for this salivary gland neoplasm, mainly due to its rare occurrence. Despite the differences in available treatment options for patients with sinonasal epithelial-myoepithelial carcinoma among the case reports, majority of the patients underwent wide surgical excision through open and/ or endoscopic approach with secure clear margins. The efficacy of adjuvant radiation therapy and/ or chemotherapy remains unclear because of its relatively indolent biological behavior.^[5]^ The tumor size of < 4 cm, absence of regional nodal or distant metastases, patient age of < 80 years at diagnosis, and undergoing surgical treatment were influencing factors of the overall survival benefit.^[2,6]^ Reports have suggested the relationships between the histopathologic findings and clinical course of salivary gland malignancies.^[13,14]^ Wakasaki et al reported that myoepithelial carcinoma originating from a minor salivary gland and low proliferation index of Ki-67 labeling indicate favorable prognosis in the patients.^[14]^ Considering the results of previous studies on prognostic factors of such salivary neoplasms, the epithelial-myoepithelial carcinoma originating in the nasal septum of our study had favorable prognostic factors (primary site, tumor size, absence of regional or distant metastases, age of < 80 years, and low Ki-67 labeling index (3.5%)), which indicates that endonasal endoscopic tumor excision alone without adjuvant therapy may be the effective treatment of choice. Despite accumulating evidence for the risk factors of epithelial-myoepithelial carcinoma, studies including larger number of cases are necessary to determine effective treatment strategies.

## 4 Conclusion

We described a rare case of epithelial-myoepithelial carcinoma arising from the nasal septum in a 75-year-old female patient. The patient had good prognostic factors determined in previous studies, and achieved complete treatment-response. Further accumulation of cases and long-term follow-up data are needed to elucidate the pathophysiology and prognosis of epithelial-myoepithelial carcinoma.

## Author contributions

**Data curation:** Sun Mok Ha, Seung Won Paik, Hyun Jong Jeon.

**Formal analysis:** Sun Mok Ha, Seung Won Paik, Hyun Jong Jeon.

**Investigation:** Hyun Jong Jeon.

**Methodology:** Hui Joon Yang.

**Supervision:** Dong-Joon Park, Chi Sang Hwang.

**Validation:** Chi Sang Hwang.

**Visualization:** Hui Joon Yang, Chi Sang Hwang.

**Writing – original draft:** Young Sub Lee, Chi Sang Hwang.

**Writing – review & editing:** Dong-Joon Park, Chi Sang Hwang.
